# Study of environmental data from vehicle’s sensors and its aplicability to complement climate mapping from automatic meteorological stations and assess covid-19 impact

**DOI:** 10.1186/s42467-021-00013-y

**Published:** 2021-10-15

**Authors:** Philippe Cedraz Lopes, Juliana Carla Santos da Silva, Lílian Lefol Nani Guarieiro, Davidson Martins Moreira

**Affiliations:** SENAI CIMATEC, Avenida Orlando Gomes, 1845. Zip Code, Salvador, BA 41650-010 Brazil

**Keywords:** Automatic meteorological stations, Connected vehicles, Weather data, Data mesh, COVID-19

## Abstract

An evolution of smart and connected cars allows the advancement of smart cities and new business models for automakers. The main objective of this article was to understand the capability of Brazilian vehicles to collect meteorological data, through an observational approach of vehicle technologies and an applied study of automatic weather stations. In 2020, when the world was affected by the COVID-19 pandemic, many studies were conducted in order to find a possible relationship between these meteorological data and the incidence of the novel coronavirus. Through this study, meteorological variables that are collected by the stations, as well as vehicles, were compared in order to evaluate the potential of data combination, in addition to the analysis of the influence of these variables in pandemic cases like COVID-19. In this context, it was understood the vehicle’s advancement as a mobile sensor and the usage of vehicle’s data as a tool for a better understanding of the COVID-19 pandemic.

## Introduction

The Internet of Things is based on the integration of a series of connected devices, in some cases, with embedded sensors capable of collecting data in real-time. It allows a new approach for solving complex systems and data analysis, for instance, house automation, intelligent traffic management systems, and environmental monitoring systems [1].

Nowadays, several monitoring devices can be deployed in a large area to collect data in real-time according to the needs of the application. New business models have emerged from the data monetization of several connected devices [2]. Companies aim to have products and services focused on the consumer, based on a deep analysis of real-time information, data mining and cluster analysis, and others. The same strategy can be applied to public departments as well, which aim to collect essential information for better country management. In the segment of meteorological forecast, for instance, one of the key devices being used for monitoring Brazil’s climate conditions is the AMS (Automatic Meteorological Stations), which are spread around the country area in order to collect data from several cities and process all information for weather forecasts [3].

Public departments and private companies are increasingly looking to use data that allows better management of urban mobility and city development planning [4]. These institutions tend to use relevant information from connected platforms as a database to analyze the profile and behavior of people and urban mobility. For car manufacturers, new business models are being raised from vehicle data on the road as part of the enterprise value strategy. For instance, vehicles are capable of measuring several environmental data around their current location for their own functionality [5].

Currently, there are companies that use this type of database as a business model by transforming it into actionable insights, converting it into useful information for each type of company. The American startup ClimaCell© has been expanding in this market precisely with this approach. Among the companies in its list of clients, there are JetBlue, Uber, and Ford, among others. In addition to providing data for the private sector, ClimaCell’s database is also used in scientific studies. In recent research about how air quality directly impacts COVID-19 [6], it was used ClimaCell’s global air quality data set [7, 8].

Other climate database platforms have contributed worldwide to a better understanding of the correlation between COVID-19 and weather, such as Weather Source©, which opened its API (Application Programming Interfaces) to researchers all over the world when it visualized potential in this line of research [9].

One of the most recent projects in this research area is led by a group of stakeholders from industry and universities from several countries (between Europe and Asia) that aims to obtain real-time weather information on highways from a multimodal network that uses the vehicle’s location. The main objective of this research is to create a service in a near future that uses not only the traditional climate measurement system of static physical stations, but also a wide fleet of connected vehicles. The data can be collected by a trigger of events that happens at a certain moment or through a time series of data from embedded sensors. In the second instance, the data can be processed on a central server for vehicle’s behavior analysis, and projected on maps of a proprietary platform called “Be-Mobile’s Mobility Platform”. In parallel, the data can be trained and validated by a machine learning algorithm. Finally, the platform intends to send alerts to partners associated with the project, as well as drivers and government agencies [10].

In this initial phase of the research, the authors analyzed the quality of vehicle data obtained from three vehicles based on the installation of external sensing units coupled to the vehicle. The platform of this project initially aims to use only 30 vehicles in a limited way for initial tests and validation but intents on having a wider fleet of vehicle in the next project development phase. The authors did not compare all the meteorological variables from the vehicles to the stationary weather stations, since the main objective was to validate the quality of vehicle data.

**Fig. 1:**
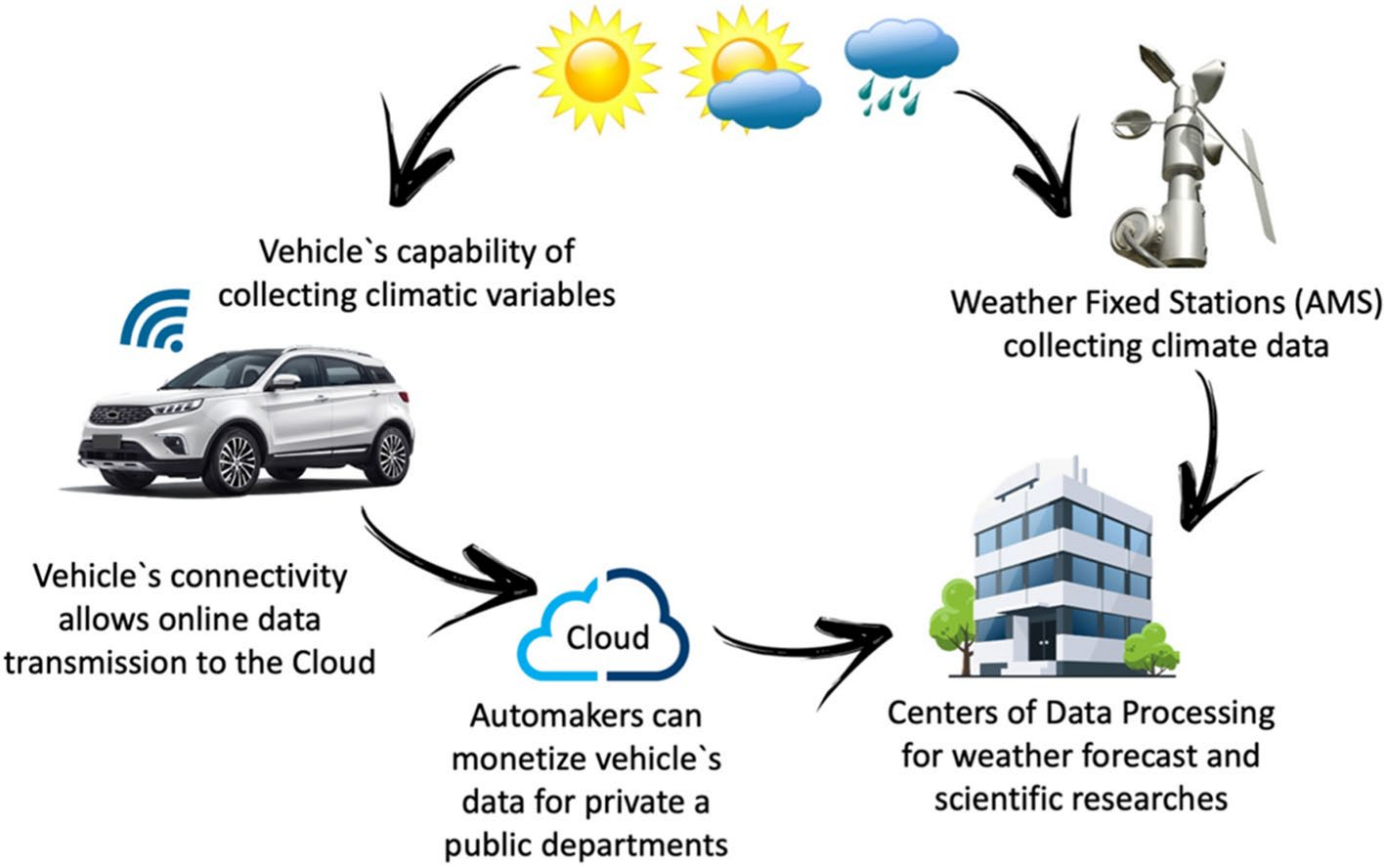
Study case diagram overview

Therefore, this study aimed to understand vehicle technologies and their ability to collect environmental data, comparing it with data collection methods from stationary weather stations and the COVID-19 pandemic. The summary illustration of this article is presented in Fig. 1. The central idea is that meteorological data can be collected by connected vehicles and weather stations in order to have a higher spatial resolution and all the data would be processed for weather forecast in a single data center.

## Methodology

The methodology for the elaboration of this review article had an observational purpose. The following question was observed through obtained data: how can automakers expand the vehicle’s usage as an essential element for smart cities when it comes to collecting climate data?

The relevance of this research was shown in the exploration of the vehicle’s capacity to contribute to the cities’ development sustainability. In addition, this study has the potential of providing important meteorological data, which can be used as a tool for COVID-19 research. As reference material for the analysis regarding COVID-19, recent scientific and statistical studies were collected from researchers all around the world, for a better understanding of the scenario.

## Results and discussion

### Environmental sensors in Brazilian vehicles

Nowadays, several automakers present a series of vehicle technologies that allow comfort, safety, and convenience to improve users’ experience [5]. Among these, vehicle sensors are capable of identifying rain on the windshield and measuring external ambient parameters like temperature, atmospheric pressure, relative humidity of the air, the intensity of solar radiation, and the intensity of external luminosity and link all these variables with the vehicle geolocation based on the Global Positioning System (GPS). Currently, all these embedded sensors aim to provide information and data only for the local vehicle application.

Rain sensors use the principle of reflection of the windshield to identify, in some levels, rain intensities, allowing the automatic activation of the windshield wipers with variable speeds [11]. The OAT (Outside Air Temperature) sensor, relative humidity, and solar radiation are used in the air conditioning controls and its transfer function in order to adjust the temperature of the internal compartment [11, 12]. The main objective of measuring the intensity of external light (from the environment and other vehicles) is to automatically activate the headlights and adjust the direction of the light projection, being an item of convenience and safety for the driver. Since all the sensors are designed for automotive applications, they have high reliability and operating precision.

These vehicle sensors that monitor external vehicle variables enable the vehicle perspective as a mobile data collection unit that has high territorial coverage. Complementary to the sensing system, automakers started to introduce embedded modems in their vehicles, allowing connectivity to external servers (through Wi-Fi and 3G / 4G connection) and leveraging the ability of smart cars to transmit high precision data in real-time [13]. The transmission can happen in real-time, or it can be scheduled for specific times.

### Connected vehicles capacity

Vehicles are being designed with the ability to connect to the internet and have a series of data being transmitted to major automaker servers. From the point where vehicles move constantly in large urban centers, automakers are now expanding their perspective to see vehicles not only as a mobility mechanism but also as a way of obtaining data from users and cities [13].

Vehicles are now being considered as connected mobile sensors, capable of extending the power of smart cities by mapping data around an entire city. Data such as car flow and schedules, meteorological conditions, and pollutant status can be easily obtained in an entire urban area from connected cars through several communication strategies, like V2I (Vehicle To Infrastructure), V2V (Vehicle To Vehicle), V2P (Vehicle to People), and V2N (Vehicle To Network), among others [14]. Therefore, vehicles as mobile sensors distributed in cities will assist the expansion of smart cities, forming a heterogeneous system capable of collecting various pieces of information from large urban centers. The expansion of the user experience and automotive technologies allow automakers to innovate and explore new scientific research and develop new connected technologies for several segments: academic research, industry, hospitals, and government departments.

### Automatic meteorological stations

**Fig. 2:**
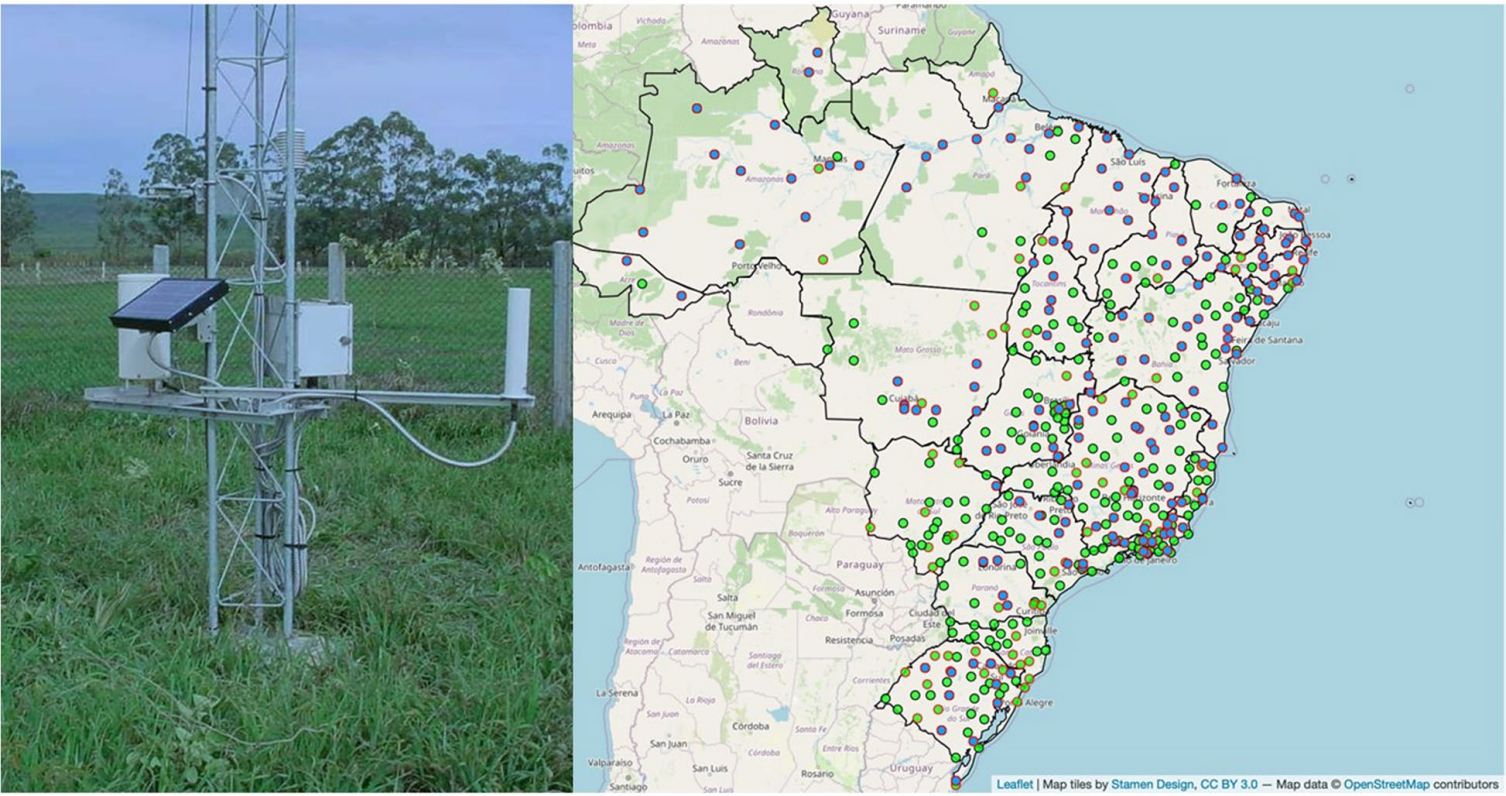
Example of an Automatic Meteorological Stations (left) and distribution of automatic weather stations across Brazilian territory (right) [3, 15]

Nowadays, the most traditional method of collecting weather data is through AMS, which are distributed throughout Brazil, as shown in Fig. 2. According to the Brazilian National Meteorological Institute (*Instituto Nacional de Meteorologia* - INMET), this system is responsible for collecting information such as temperature, humidity, atmospheric pressure, precipitation, wind direction and speed, and solar radiation every minute, regarding the area in which they are located [3]. Every hour this information is compiled and structured so that it can then be sent to INMET headquarters, where it will be validated and then stored in a database.

The stations are composed of four subsystems: data collection, data storage, energy, and communication. The data collection subsystem is a network of sensors equipped to measure certain meteorological parameters. For stations that are not fully automated, manual sensors can be installed in order to measure additional variables such as total cloudiness, cloud height, cloud base height, and horizontal visibility [3].

Moreover, the collected data is recorded in the storage subsystem, using a low-power processor. These values are stored in a memory unit for a predetermined time. The power subsystem is equipped with photovoltaic panel modules and is responsible for supplying the necessary energy for the operation of the station’s instruments, making it independent of external electrical energy [3]. Finally, the communication subsystem transmits the information registered at the AMS to INMET’s headquarters, via satellite, or cell phone networks. The transmission can happen in real-time, or it can be scheduled for specific times.

### Correlation between automatic meteorological stations and smart vehicles

In the Brazilian scenario, the estimate for the fleet of conventional automobiles and medium and large commercial pick-ups are approximately 43.2 million spread across the national territory. Approximately 2.8 million new cars were produced in 2019 and this number grows year by year, according to the National Association of Vehicle Manufacturers (*Associação Nacional dos Fabricantes de Veículos Automotores* - ANFAVEA) [16]. There are 775 automatic and conventional meteorological stations in the Brazilian territory registered by INMET, which represent 0.028% of the number of cars produced only in 2019. Thus, in quantitative analysis, it is possible to understand the dimension of a vehicle’s dispersion compared to the number of meteorological stations in the Brazilian territory.

Regarding the variables of the data obtained, AMS has a data collection subsystem capable of measuring at least 18 meteorological parameters [3], distributed in 8 different categories of information, as shown in Table 1. Through comparison analysis with current vehicles’ subsystems, conventional automobiles do not have the ability to measure seven meteorological parameters, related to the dew point temperatures; speed, direction, and wind intensity; and also the rainfall volume accumulated in the period. Therefore, AMS have a greater capacity to collect meteorological data in comparison to smart vehicles.

**Table 1:** Comparison between automatic meteorological stations and smart vehicles [3, 5, 11, 12]

Meteorological Variable Category	Specific Meteorological Variable Description	Ams’ measurement capability	Vehicle’s measurement capability
**Temperature**	Current Air Temperature (°C)	X	Real-Time Temperature
	Maximum Air Temperature (°C)	X	
	Minimum Air Temperature (°C)	X	
**Humidity**	Current Relative Air Humidity (%)	X	Real-Time Humidity
	Maximum Relative Air Humidity (%)	X	
	Minimum Relative Air Humidity (%)	X	
**Dew Point**	Current Dew Point Temperature (°C)	X	–
	Maximum Dew Point Temperature (°C)	X	–
	Minimum Dew Point Temperature (°C)	X	–
**Atmospheric Pressure**	Current Atmospheric Air Pressure (hPa)	X	Real-Time Atmospheric Pressure
	Maximum Atmospheric Air Pressure (hPa)	X	
	Minimum Atmospheric Air Pressure (hPa)	X	
**Wind**	Current Wind Speed (m/s)	X	–
	Wind Direction (°)	X	–
	Wind Gust Intensity (km/h)	X	–
**Radiation**	Solar Radiation (kJm^2^)(W)	X	X
**Rain Level**	Accumulated Precipitation in the Period (mm)	X	–
	Relative Rain Intensity Level	X	X
**GPS Coordinate**	Latitude	X	X
	Longitude	X	X
	Altitude (m)	X	X
**Time**	Collection Date [day/month/year]	X	X
	Collection Time [hour/minute/second]	X	X

In terms of expansion of the sensing subsystem, AMS platforms are modular and allow the addition of new sensors at any station, depending on the need identified by the stakeholders involved [3]. On the other hand, automakers can add new sensors to their vehicles by launching new models through technical and economic feasibility. The adaptation period of both platforms was not analyzed in this study.

### Correlation between the above-mentioned climate variables and COVID-19

In December 2019, the world became aware of a new respiratory pathology with unknown nature, which originated in the city of Wuhan, China. In January 2020, the World Health Organization (WHO) declared a warning of Public Health Emergency of International Concern, regarding the disease called COVID-19 caused by the beta-coronavirus Sars-Cov-2 [17]. According to the epidemiological bulletin, in February 2021, 103,362,039 cases were confirmed globally. Furthermore, the main form of transmission is through respiratory contact [18].

Given the scenario of health crisis and uncertainty regarding the properties of the virus and the disease, several studies have since been conducted in order to understand the nature of this pandemic. One of the topics widely addressed by researchers around the world is the possible relationship between the rate of transmission and the severity of the disease, compared to meteorological factors in each region.

Although there is research that does not present a coherent relationship in places like China, Spain, and the United States, in most research that analyzes specific areas of study [19], positive correlations were pointed out between the above-mentioned variables and analyzed through Table 2.

**Table 2:** Impact of the above-mentioned meteorological variables on COVID-19

		Relationship with COVID-19	Record	Site
**Climate Variable**	**Temperature**	X	[20]	USA
			[21]	Global
	**Humidity**	X	[22]	Iran
			[21]	Global
			[19]	Brazil
	**Dew Point**	–	–	–
	**ATM Pressure**	–	–	–
	**Wind**	X	[22]	Iran
	**Radiation**	X	[22]	Iran
	**Air Quality**	X	[20]	USA
	**Rain Level**	X	[23]	Global

In a study carried out in New York City (United States of America), a statistical relationship was observed between average temperature, minimum temperature, air quality and the cases of COVID-19 in the city [20]. A survey conducted in the Middle East pointed out that, in Iran, there were “areas with low values of wind speed, humidity, and solar radiation exposure to a high rate of infection that support the virus’s survival” [22]. In addition, in a review of several studies on the topic around the world, it was shown that:

*The findings of this review suggest that there is a significant association between both temperature and humidity and COVID-19 incidence [...]The significant effect of temperature and humidity on COVID-19 incidence is consistent with findings in earlier studies on airborne respiratory viruses, including SARs, influenza, respiratory syncytial virus (RSV) and MERs* [21]*..*

In a study conducted in Brazil analyzing data from Brazilian capitals, among the meteorological variables studied, a correlation was found between the average humidity of the air and the cases of the COVID-19 pandemic [19].

**Fig. 3:**
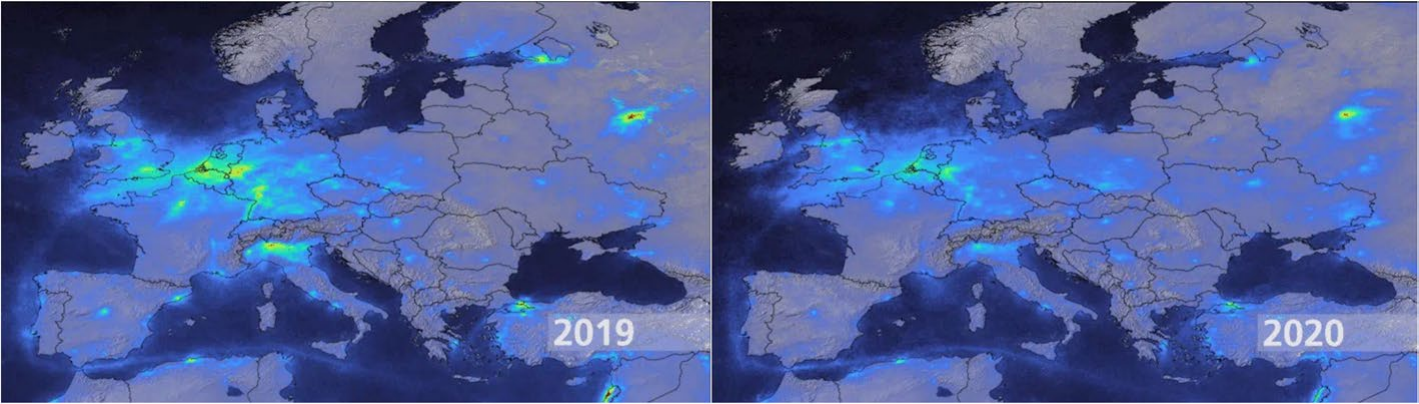
Comparison of nitrogen dioxide emissions over Europe between March and April 2019 (left) and 2020 (right) [24]

However, in addition to the influence of meteorological variables in the COVID-19 pandemic, the opposite phenomenon can also be observed: the scenario caused by the virus directly impacted global climate indices. According to IEEE Spectrum [24]: *“Since the early days of the COVID-19 pandemic, scientists and civilians on the ground have observed a sharp improvement in air quality, especially over quarantined regions.”* As shown in Fig. 3, German Aerospace Center Remote Sensing Technology Institute research shows a healthier atmosphere between 2019 and 2020.

In view of the study presented above, it can be concluded that the combination of these potential results can lead to the benefit of the population and the health system. Since the data provided by smart cars has more resolution and are more accurate, meteorological data platforms using this kind of source represent a positive contribution not only to ordinary applications like weather forecasts, agriculture, sailing, and others, but also health applications like disease prevention and diagnosis, such as COVID-19.

## Conclusion

In this article, the sensorial capacity of conventional Brazilian automobiles for the collection of meteorological data and their connectivity subsystem was analyzed. The addressed theoretical foundation allowed us to understand which variables can be collected from vehicles and analyze how the automakers can connect this real-time information through a data grid. In parallel, a study was carried out on the AMS also addressing the capacity of its data collection subsystem. The main objective was to carry out a comparative analysis of the sensory capacity between Brazilian vehicles and AMS. Therefore, the main conclusion is that automakers have the infrastructure and the control of the vehicle’s fleet that is capable of collecting meteorological data with high territorial coverage towards new advances in the scientific climate area.

This study is important not only for weather institutes and universities, but also for the growth of automotive industry services that can leverage the potential of vehicle’s data using artificial intelligence for weather forecast. The perspective around the meteorological data processing it is also relevant in the COVID-19 context, given that half of the climate variables presented in the comparison can be related to the incidence of the disease, as shown in Table 2. As presented in section 3.4, analyzed Brazilian vehicles can collect 11 meteorological parameters while AMS currently collect 18 variables, which makes automobiles a high potential mobile device for relevant data collection. In parallel, with the connectivity power established by the automakers in recent years, automobiles gain the ability to transmit data and reach a territorial coverage superior to AMS thereby increasing the density of the collected data and the granularity of information, which can contribute to a better understanding of the climate variables’ relation with COVID-19 by making research with more accurate data possible.

However, in order to leverage the automakers’ enterprise value as data providers, investments in the creation of private digital platforms and data sharing are necessary. Once the automakers analyze the reliability of the mesh data and understand the real needs of meteorological institutes, public departments, and other private companies, new scientific models will be adapted, allowing technological-scientific advancement through the use of artificial intelligence. As suggestions for future research, a deep analysis of private data sharing based on Brazilian legal requirements should be performed, and the use of artificial intelligence and machine learning to evolve existing mathematical models linked to weather forecasting based on the massive availability of vehicle data should be studied.

## Data Availability

Not applicable.

## Abbreviations

AMS: Automatic Meteorological Stations
API: Application Programming Interfaces
GPS: Global Positioning System
OAT: Outside Air Temperature
V2I: Vehicle To Infrastructure
V2V: Vehicle To Vehicle
V2P: Vehicle to People
V2N: Vehicle To Network
INMET: Instituto Nacional de Meteorologia
ANFAVEA: Associação Nacional dos Fabricantes de Veículos Automotores
WHO: World Health Organization
